# Impact of Different Physical Exercises on the Expression of Autophagy Markers in Mice

**DOI:** 10.3390/ijms22052635

**Published:** 2021-03-05

**Authors:** Ana P. Pinto, Alisson L. da Rocha, Bruno B. Marafon, Rafael L. Rovina, Vitor R. Muñoz, Lilian E. C. M. da Silva, José R. Pauli, Leandro P. de Moura, Dennys E. Cintra, Eduardo R. Ropelle, Adelino S. R. da Silva

**Affiliations:** 1 Postgraduate Program in Rehabilitation and Functional Performance, Ribeirão Preto Medical School, University of São Paulo (USP), Avenida Bandeirantes, 3900, Monte Alegre, Ribeirão Preto 14040-907, Brazil; anapp_5@hotmail.com (A.P.P.); alissonldarocha@gmail.com (A.L.d.R.); 2 School of Physical Education and Sport of Ribeirão Preto, University of São Paulo (USP), Avenida Bandeirantes, 3900, Monte Alegre, Ribeirão Preto 14040-907, Brazil; bruno.marafon@usp.br (B.B.M.); rafael.rovina@usp.br (R.L.R.); 3 Laboratory of Molecular Biology of Exercise (LaBMEx), School of Applied Sciences, University of Campinas (UNICAMP), Rua Pedro Zacarias, 1300, Limeira 13484-350, Brazil; vitor.munoz93@gmail.com (V.R.M.); rodrigopaulifca@gmail.com (J.R.P.); leandropereiram@hotmail.com (L.P.d.M.); dcintra@yahoo.com (D.E.C.); eduardoropelle@gmail.com (E.R.R.); 4 Department of Ophthalmology, Otorhinolaryngology, and Head and Neck Surgery, School of Medicine of Ribeirão Preto, University of São Paulo (USP), Avenida Bandeirantes, 3900, Monte Alegre, Ribeirão Preto 14040-907, Brazil; lilianeslaine@fmrp.usp.br

**Keywords:** colchicine, autophagic flux, time course, gastrocnemius, heart, liver

## Abstract

Although physical exercise-induced autophagy activation has been considered a therapeutic target to enhance tissue health and extend lifespan, the effects of different exercise models on autophagy in specific metabolic tissues are not completely understood. This descriptive investigation compared the acute effects of endurance (END), exhaustive (ET), strength (ST), and concurrent (CC) physical exercise protocols on markers of autophagy, genes, and proteins in the gastrocnemius muscle, heart, and liver of mice. The animals were euthanized immediately (0 h) and six hours (6 h) after the acute exercise for the measurement of glycogen levels, mRNA expression of *Prkaa1*, *Ppargc1a*, *Mtor*, *Ulk1*, *Becn1*, *Atg5*, *Map1lc3b*, *Sqstm1*, and protein levels of Beclin 1 and ATG5. The markers of autophagy were measured by quantifying the protein levels of LC3II and Sqstm1/p62 in response to three consecutive days of intraperitoneal injections of colchicine. In summary, for gastrocnemius muscle samples, the main alterations in mRNA expressions were observed after 6 h and for the ST group, and the markers of autophagy for the CC group were increased (i.e., LC3II and Sqstm1/p62). In the heart, the Beclin 1 and ATG5 levels were downregulated for the ET group. Regarding the markers of autophagy, the Sqstm1/p62 in the heart tissue was upregulated for the END and ST groups, highlighting the beneficial effects of these exercise models. The liver protein levels of ATG5 were downregulated for the ET group. After the colchicine treatment, the liver protein levels of Sqstm1/p62 were decreased for the END and ET groups compared to the CT, ST, and CC groups. These results could be related to diabetes and obesity development or liver dysfunction improvement, demanding further investigations.

## 1. Introduction

Regular exercise improves glucose homeostasis, skeletal muscle mass, cardiovascular fitness, and liver health. Physical activity initiates many events, leading to increased oxidative stress, energetic imbalance, unfolding proteins, and intracellular calcium, which can induce autophagy activation [1]. Muscle contraction activates molecular messengers, such as reactive oxygen species (ROS), nicotinamide and adenine dinucleotide (NAD+), calcium, and adenosine monophosphate (AMP). These messengers signal downstream cascades, leading up to an autophagic response to restore homeostasis [1]. When energy supply and demand are balanced, the mechanistic target of rapamycin (mTOR) negatively regulates autophagy by the phosphorylation and inhibition of unc-51-like autophagy activating kinase 1 (ULK1) [2]. When the energy demand increases, the AMP:ATP ratio also increases, activating AMP-activated protein kinase (AMPK) and inhibiting mTOR activity. AMPK phosphorylates ULK1, at different phosphorylation sites, and leads to autophagy induction. During exercise, the increase in NAD+ and ROS production generates the activation of AMPK, as well as p38 mitogen-activated protein kinase (p38MAPK) and Sirtuin-1 (SIRT1) [3]. These molecules, in turn, lead to the activation of the autophagic pathway. AMPK upregulates peroxisome proliferator-activated receptor gamma coactivator 1-alpha (PGC-1α) activity. When active, PGC-1α co-activates transcription factors in the nucleus, generating an increase in autophagy and lysosomal genes.

**Figure 1:**
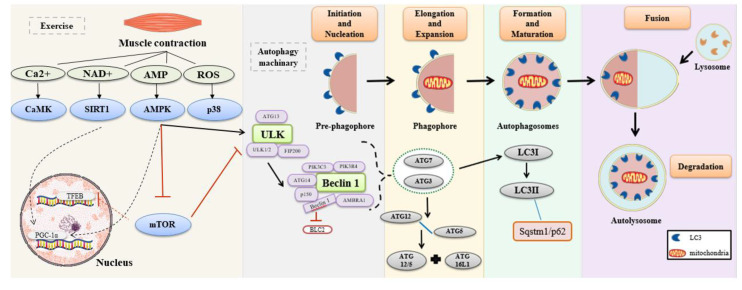
Autophagy pathway process. Muscle contraction increases the levels of Ca2+, adenine dinucleotide (NAD+), adenosine monophosphate (AMP), and reactive oxygen species (ROS). These molecules activate their downstream effectors, which initiates autophagy activation. AMP-activated protein kinase (AMPK) activates autophagy induction complex by phosphorylation of unc-51-like autophagy activating kinase 1 (ULK1). The process begins with the formation of autophagic protein complex ULK (ULK1/2 + ATG13 + FIP200). Thereby, the mechanism initiation and activation of a second complex occur, named Beclin 1 (PIK3C3 P1K3R4+ Beclin 1 + ATG14 + p150 + Ambra 1). In this stage, Beclin 1 is dissociated from BCL-2, and this dissociation is responsible for the conduction of the Beclin 1 complex to the phagophore membrane, starting the nucleation. In sequence, activation of ATG7 occurs. ATG7 and ATG10 mediated the binding of ATG12-ATG5 conjugate to an ATG16L1 dimer. The ATG7 and ATG3 proteins generate the phosphatidylethanolamine (PE)-conjugation of LC3I, creating the LC3II, and producing the closure of phagophore in autophagosomes. The LC3II interacts with Sqstm1/p62. In sequence, the fusion occurs with the lysosome to form the autolysosome for degradation [29,30]. Solid and dashed black arrows activate the molecule or the complex. T red arrow inhibit the molecule or the complex

Autophagy is an evolutionarily conserved lysosomal degradation process with a crucial role in health and disease [4], which involves several steps, including induction, phagophore elongation and expansion, and formation and maturation of the autophagosome, which fuses with the lysosomes to form the autolysosomes, and cargo degradation [5]. Autophagy is essential for physiological adaptions to exercise and consists of providing energy substrates [6]. Figure 1 summarizes the autophagy pathway process.

Autophagy induction and inhibition are related to the activation of AMPK and mTOR, respectively [7]. While the AMPK/*PGC-1α* pathway is related to endurance exercise, the protein kinase B (Akt)/mTOR pathway is mainly related to strength exercises [8]. However, in response to concurrent exercise (a combination of strength and endurance exercises), both proteins can be activated [9,10]. The majority of investigations about the effects of exercise on the autophagic pathway in multiple tissues used endurance exercise protocols [11,12,13,14,15,16,17,18,19]. The type of exercise and its intensity can differently upregulate autophagy [20]. The studies evaluating the impact of strength [21] or concurrent exercise [22] on autophagy are minimal and only analyzed skeletal muscle samples.

Autophagy activation has been considered a therapeutic target to improve tissue health and induce lifespan extension [23,24,25]. In the heart, autophagy is essential to myocyte function and survival [26]. In the liver, autophagy is vital to the adaptation to starvation through the initiation of glycogenolysis, lipolysis, and protein catabolism [27]. Although endurance exercise leads to autophagy in specific metabolic tissues [28], the effects of strength or concurrent exercise on autophagy in the heart and liver are unknown. In addition, the comparison among different physical exercise protocols on the autophagic pathway activation has not been explored. Thus, we compared the acute effects of endurance, exhaustive, strength, and concurrent physical exercise protocols on autophagy genes and proteins, as well as markers of autophagic flux in mice metabolic tissues. 

## 2. Results

### 2.1. Body Weight and Glucose Concentrations

Table 1 shows that the body weight and basal glucose were not different between the experimental groups. The ET and CC presented a reduction in the post-exercise glucose compared to the basal glucose. The delta variation of the ST was higher than the ET.

### 2.2. Effects of Acute Physical Exercise Protocols on Gastrocnemius Muscle

**Figure 2:**
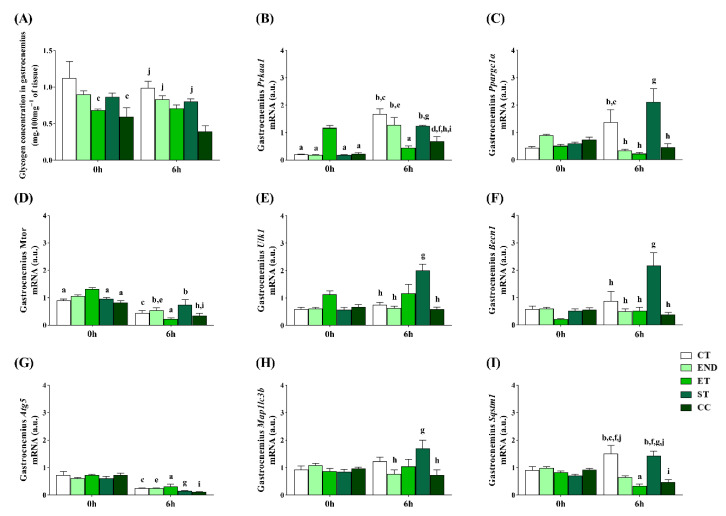
Glycogen concentrations in the gastrocnemius muscle (**A**). Gastrocnemius mRNA levels of: *Prkaa1* (**B**), *Ppargc1a* (**C**), *Mtor* (**D**), *Ulk1* (**E**), *Becn1* (**F**), *Atg5* (**G**), *Map1lc3b* (**H**), *Sqstm1* (**I**). Data correspond to the mean ± SE of *n* = 5 mice/group. ^a^ *p* ≤ 0.05 vs. ET at 0 h; ^b^ *p* ≤ 0.05 vs. ET at 6 h; ^c^ *p* ≤ 0.05 vs. CT at 0 h; ^d^ *p* ≤ 0.05 vs. CT at 6 h; ^e^ *p* ≤ 0.05 vs. END at 0 h; ^f^ *p* ≤ 0.05 vs. END at 6 h; ^g^ *p* ≤ 0.05 vs. ST at 0 h; ^h^ *p* ≤ 0.05 vs. ST at 6 h; ^i^ *p* ≤ 0.05 vs. CC at 0 h; ^j^ *p* ≤ 0.05 vs. CC at 6 h. Control (CT: sedentary); Endurance (END: submitted to the endurance protocol); Exhaustive (ET: submitted to the exhaustive protocol); Strength (ST: submitted to the strength protocol); Concurrent (CC: submitted to the concurrent protocol).

The glycogen concentrations in the gastrocnemius at 0 h were lower for the ET and CC than the CT. At 6 h, the CT, END, and ST had higher levels than the CC. (Figure 2A). The *Prkaa1* mRNA at 0 h was lower for the CT, END, ST, and CC than the ET. At 6 h, the CT, END, and ST had higher *Prkaa1* mRNA than the ET, and the CC was lower than the CT, END, and ST groups. Compared to 0 h, the CT, END, ST, and CC had higher *Prkaa1* mRNA at 6 h, while the ET had lower levels (Figure 2B).

The *Ppargc1a* mRNA at 0 h was not different between the experimental groups. At 6 h, the CT, END, ET, and CC had lower *Ppargc1a* mRNA than the ST, and the CT was higher than the ET. Compared to 0 h, the CT and ST had higher *Ppargc1a* mRNA at 6 h (Figure 2C). The *Mtor* mRNA at 0 h was lower for the CT, ST, and CC than the ET group. At 6 h, the END and ST had higher *Mtor* mRNA than the ET, and the CC was lower than the ST. Compared to 0 h, the CT, END, ET, and CC groups had lower *Mtor* mRNA at 6 h (Figure 2D). 

The *Ulk1* and *Becn1* mRNA at 0 h were not different between the experimental groups (Figure 2E,F). At 6 h, the CT, END, and CC had lower *Ulk1* mRNA than the ST. Compared to 0 h, the ST had higher *Ulk1* mRNA at 6 h (Figure 2E). At 6 h, the CT, END, ET, and CC had lower *Becn1* mRNA than the ST. Compared to 0 h, the ST had higher *Becn1* mRNA at 6 h (Figure 2F). The *Atg5* mRNA at 0 and 6 h was not different between the experimental groups. Compared to 0 h, the CT, END, and ST had lower *Atg5* mRNA at 6 h, while the ET and CC had lower levels (Figure 2G). 

The *Map1lc3b* and *Sqstm1* mRNA at 0 h was not different between the experimental groups (Figure 2H,I). At 6 h, the END and CC had lower *Map1lc3b* mRNA than the ST. Compared to 0 h, the ST had higher *Map1lc3b* mRNA at 6 h (Figure 2H). At 6 h, the CT and ST had higher *Sqstm1* mRNA than the END, ET, and CC. Compared to 0 h, the CT and ST had higher *Sqstm1* mRNA at 6 h, while the ET and CC had lower levels (Figure 2I). 

**Figure 3:**
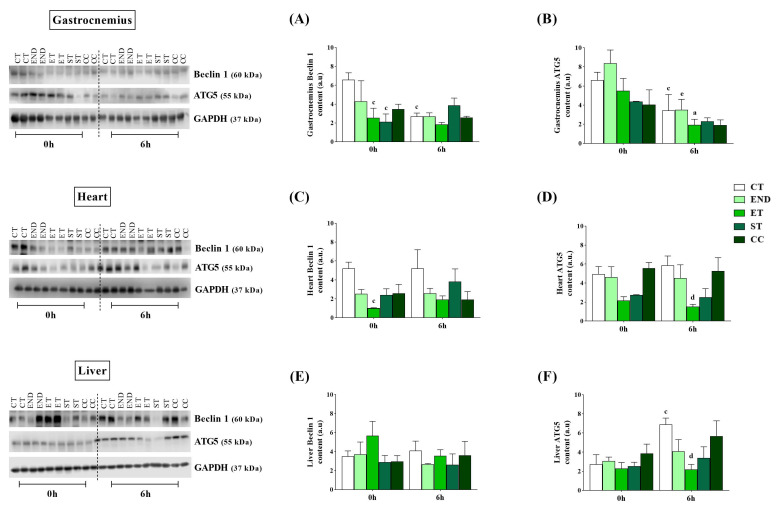
Gastrocnemius protein levels of Beclin 1 (**A**) and ATG5 (**B**). Heart protein levels of Beclin 1 (**C**) and ATG5 (**D**). Liver protein levels of Beclin 1 (**E**) and ATG5 (**F**). Data correspond to the mean ± SE of *n* = 5 mice/group. ^a^ *p* ≤ 0.05 vs. ET at 0 h; ^c^ *p* ≤ 0.05 vs. CT at 0 h; ^d^ *p* ≤ 0.05 vs. CT at 6 h; ^e^ *p* ≤ 0.05 vs. END at 0 h; Control (CT: sedentary); Endurance (END: submitted to the endurance protocol); Exhaustive (ET: submitted to the exhaustive protocol); Strength (ST: submitted to the strength protocol); Concurrent (CC: submitted to the concurrent protocol). Ponceau with full bands and kDa are in Supplementary File 1.

The Beclin 1 protein level was higher for the CT group than the ET and ST groups (Figure 3A). The Beclin 1 at 6 h was not different between the experimental groups. Compared to 0 h, the CT had a lower Beclin 1 protein level at 6 h (Figure 3A). The ATG5 protein level was not different between the experimental groups at 0 h and 6 h (Figure 3B). Compared to 0 h, the CT, END, and ET groups had lower protein levels at 6 h (Figure 3B). 

**Figure 4:**
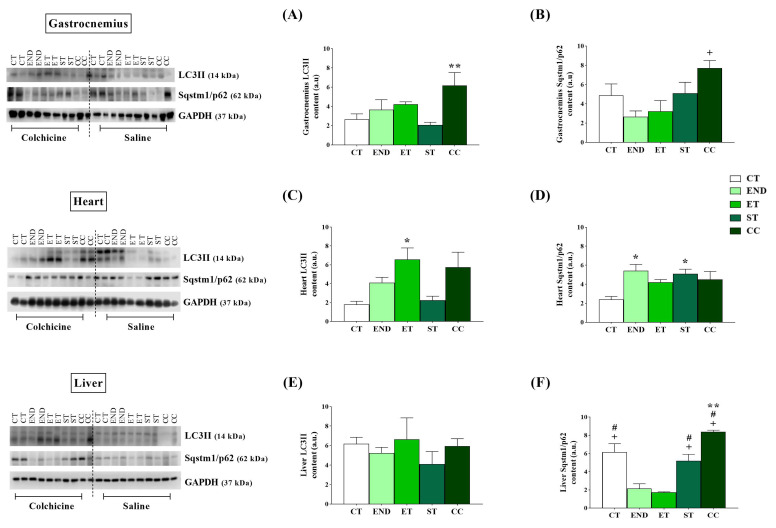
Gastrocnemius protein levels of LC3II (**A**) and Sqstm1/p62 (**B**). Heart protein levels of LC3II (**C**) and Sqstm1/p62 (**D**). Liver protein levels of LC3II (**E**) and Sqstm1/p62 (**F**), after colchicine treatment. The mean colchicine values were subtracted from the vehicle values (saline) of corresponding protocols for autophagic flux calculation. Data correspond to the mean ± SE of *n* = 5 mice/group. * *p* ≤ 0.05 vs. CT; ^+^ *p* ≤ 0.05 vs. END; ^#^ *p* ≤ 0.05 *vs.*ET; ** *p* ≤ 0.05 vs. ST for autophagic flux. Control (CT: sedentary); Endurance (END: submitted to the endurance protocol); Exhaustive (ET: submitted to the exhaustive protocol); Strength (ST: submitted to the strength protocol); Concurrent (CC: submitted to the concurrent protocol). Ponceau with full bands and kD are in Supplementary File 2.

Regarding the markers of autophagy, the LC3II protein level was higher for the CC group compared to the ST group (Figure 4A). The Sqstm1/p62 protein level was higher for the CC group than the END group (Figure 4B).

### 2.3. Effects of Acute Physical Exercise Protocols on the Heart

**Figure 5:**
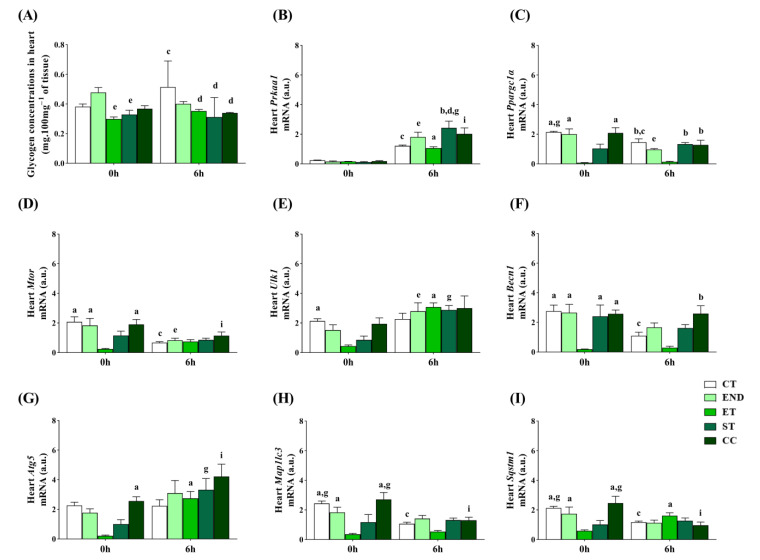
Glycogen concentrations in the heart (**A**). Heart mRNA levels of: *Prkaa1* (**B**), *Ppargc1a* (**C**), *Mtor* (**D**), *Ulk1* (**E**), *Becn1* (**F**), *Atg5* (**G**), *Map1lc3b* (**H**), *Sqstm1* (**I**). Data correspond to the mean ± SE of *n* = 5 mice/group. ^a^ *p* ≤ 0.05 vs. ET at 0 h; ^b^ *p* ≤ 0.05 vs. ET at 6 h; ^c^ *p* ≤ 0.05 vs. CT at 0 h; ^d^ *p* ≤ 0.05 vs. CT at 6 h; ^e^ *p* ≤ 0.05 vs. END at 0 h; ^g^ *p* ≤ 0.05 vs. ST at 0 h; ^i^ *p* ≤ 0.05 vs. CC at 0 h. Control (CT: sedentary); Endurance (END: submitted to the endurance protocol); Exhaustive (ET: submitted to the exhaustive protocol); Strength (ST: submitted to the strength protocol); Concurrent (CC: submitted to the concurrent protocol).

The heart’s glycogen concentrations at 0 h were lower for the ET and ST than the END. At 6 h, the END, ST, and CC had lower levels than the CT. Compared to 0 h, the CT had higher concentrations at 6 h (Figure 5A). The *Prkaa1* mRNA at 0 h was not different between the experimental groups. At 6 h, the ST had higher *Prkaa1* mRNA than the CT and ET. Compared to 0 h, the CT, END, ET, ST, and CC had higher *Prkaa1* mRNA at 6 h (Figure 5B). 

The *Ppargc1a* mRNA at 0 h was higher for the CT, END, and CC than the ET. In addition, the CT was higher than the ST at 0 h. At 6 h, the CT, ST, and CC had higher *Ppargc1a* mRNA than the ET. Compared to 0 h, the CT and END had lower *Ppargc1a* mRNA at 6 h (Figure 5C). The *Mtor* mRNA at 0 h was higher for the CT, END, and CC than the ET. At 6 h, the *Mtor* mRNA was not different between the experimental groups. Compared to 0 h, the CT, END, and CC had lower *Mtor* mRNA at 6 h (Figure 5D). The *Ulk1* mRNA at 0 h was higher for the CT than the ET. At 6 h, the *Ulk1* mRNA was not different between the experimental groups. Compared to 0 h, the END, ET, and ST had higher *Ulk1* mRNA at 6 h (Figure 5E). 

The *Becn1* mRNA at 0 h was higher for the CT, END, ST, and CC than the ET. At 6 h, the CC had higher *Becn1* mRNA than the ET. Compared to 0 h, the CT had lower *Becn1* mRNA at 6 h (Figure 5F). The *Atg5* mRNA at 0 h was higher for the CC than the ET. At 6 h, the *Atg5* mRNA was not different between the experimental groups. Compared to 0 h, the ET, ST, and CC had higher *Atg5* mRNA at 6 h (Figure 5G). The *Map1lc3b* mRNA at 0 h was higher for the CT, END, and CC than the ET. In addition, the CT and CC were higher than the ST at 0 h. At 6 h, the *Map1lc3b* mRNA was not different between the experimental groups. Compared to 0 h, the CT and CC had lower *Map1lc3b* mRNA at 6 h (Figure 5H). 

The *Sqstm1* mRNA at 0 h was higher for the CT, END, and CC than the ET. In addition, the CT and CC were higher than the ST at 0 h. At 6 h, the *Sqstm1* mRNA was not different between the experimental groups. Compared to 0 h, the CT and CC had lower *Sqstm1* mRNA at 6 h, while the ET had higher levels (Figure 5I). 

The Beclin 1 protein level at 0 h was higher for the CT group compared to the ET group (Figure 3C). The Beclin 1 at 6 h was not different between the experimental groups. The ATG5 protein level was not different between the experimental groups at 0 h (Figure 3D). At 6 h, the ATG5 protein level was higher for the CT group compared to the ET group (Figure 3D). 

Regarding the markers of autophagy, the LC3II protein level was higher for the ET group than the CT group (Figure 4C). The Sqstm1/p62 protein level was higher for the END and ST groups than the CT group (Figure 4D).

### 2.4. Effects of Acute Physical Exercise Protocols on the Liver

**Figure 6:**
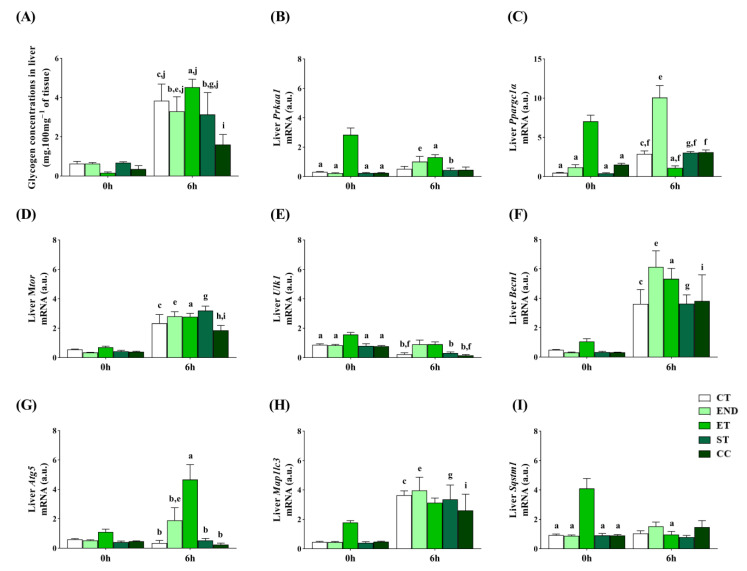
Glycogen concentrations in the liver (**A**). Liver mRNA levels of: *Prkaa1* (**B**), *Ppargc1a* (**C**), *Mtor* (**D**), *Ulk1* (**E**), *Becn1* (**F**), *Atg5* (**G**), *Map1lc3b* (**H**), *Sqstm1* (**I**). Data correspond to the mean ± SE of *n* = 5 mice/group. ^a^ *p* ≤ 0.05 vs. ET at 0 h; ^b^ *p* ≤ 0.05 vs. ET at 6 h; ^c^ *p* ≤ 0.05 vs. CT at 0 h; ^e^ *p* ≤ 0.05 vs. END at 0 h; ^f^ *p* ≤ 0.05 vs. END at 6 h; ^g^ *p* ≤ 0.05 vs. ST at 0 h; ^h^ *p* ≤ 0.05 vs. ST at 6 h; ^i^ *p* ≤ 0.05 vs. CC at 0 h; ^j^ *p* ≤ 0.05 vs. CC at 6 h; Control (CT: sedentary); Endurance (END: submitted to the endurance protocol); Exhaustive (ET: submitted to the exhaustive protocol); Strength (ST: submitted to the strength protocol); Concurrent (CC: submitted to the concurrent protocol).

The glycogen concentrations in the liver at 0 h were not different between the experimental groups. At 6 h, the CT, END, ET, and ST were higher than the CC, and the END and ST were lower than the ET. Compared to 0 h, all groups had higher values at 6 h (Figure 6A). The *Prkaa1* mRNA at 0 h was lower for the CT, END, ST, and CC than the ET. At 6 h, the ST had lower *Prkaa1* mRNA than the ET. Compared to 0 h, the END had higher *Prkaa1* mRNA at 6 h, while the ET had lower levels (Figure 6B). The *Ppargc1a* mRNA at 0 h was lower for the CT, END, ST, and CC than the ET. At 6 h, the CT, ET, ST, and CC had lower *Ppargc1a* mRNA than the END. Compared to 0 h, the CT, END, and ST had higher *Ppargc1a* mRNA at 6 h, while the ET had lower levels (Figure 6C).

The *Mtor* mRNA was not different between the experimental groups at 0 h. At 6 h, the CC had lower *Mtor* mRNA than the ST. Compared to 0 h, all groups had higher *Mtor* mRNA at 6 h (Figure 6D). The *Ulk1* mRNA at 0 h was lower for the CT, END, ST, and CC than the ET. At 6 h, the CT, ST, and CC had lower *Ulk1* mRNA than the ET, and the CT and CC were lower than the END (Figure 6E). The *Becn1* mRNA was not different between the experimental groups at 0 and 6 h. Compared to 0 h, all groups had higher *Becn1* mRNA at 6 h (Figure 6F).

The *Atg5* mRNA was not different between the experimental groups at 0 h. At 6 h, the CT, END, ST, and CC had lower *Atg5* mRNA than the ET. Compared to 0 h, the END and ET had higher *Atg5* mRNA at 6 h (Figure 6G). The *Map1lc3b* mRNA was not different between the experimental groups at 0 and 6 h. Compared to 0 h, except for ET, the other groups had higher *Map1lc3b* mRNA at 6 h (Figure 6H). The *Sqstm1* mRNA at 0 h was lower for the CT, END, ST, and CC than the ET. Compared to 0 h, the ET had lower *Sqstm1* mRNA at 6 h (Figure 6I).

The Beclin 1 was not different between the experimental groups at 0 h and 6 h (Figure 3E). The ATG5 protein levels were not different between the experimental groups at 0 h (Figure 3F). At 6 h, the ATG5 protein levels were higher for the CT group compared to the ET group (Figure 3F). Compared to 0 h, the CT had higher protein levels at 6 h (Figure 5F).

Regarding the markers of autophagy, although the LC3II protein levels were not different between the experimental groups (Figure 4E), the Sqstm1/p62 protein levels were lower for the END and ET groups compared to the CT, ST, and CC groups. In addition, the CC had higher levels compared to the ST group. (Figure 4F).

## 3. Discussion

Autophagy is a dynamic process, and its negative or positive regulation can occur in different steps, therefore autophagy markers’ responses to several experimental treatments must be accompanied by the autophagic flux evaluation [31]. To measure autophagy markers, we verified LC3II and Sqstm1/p62 protein levels in whole-cell lysates of the gastrocnemius, heart, and liver submitted to different acute physical exercise protocols following colchicine injection. In summary, for the gastrocnemius muscle, the markers of autophagy were influenced only by the concurrent physical exercise protocol. The END and ST sessions increased the heart Sqstm1/p62 protein levels after colchicine treatment. Following colchicine treatment, the hepatic Sqstm1/p62 protein levels were decreased for the END and ET groups but increased for the ST and CC groups. This positive or negative outcome should be further addressed.

### 3.1. Effects of Acute Physical Exercise Protocols on Gastrocnemius Muscle

The lower glucose and glycogen concentrations of the ET and CC compared to the CT at 0 h reinforce the metabolic challenges imposed by these acute exercise sessions.

Armstrong et al. [32] investigated the glycogen contents in the plantaris muscle in Sprague Dawley rats after running at different intensities. The authors observed that the plantaris muscle consists mostly of glycolytic fibers, and muscle glycogen decreased as the exercise intensity increased during running. Furthermore, for the first two intensities (below and in the maximal lactate steady state), no differences in the glycogen contents were observed in the glycolytic fibers. However, after the third intensity, the glycogen started to decrease [32].

In the present study, the END group’s muscle glycogen was not different from the CT group, probably because the gastrocnemius of mice contains mainly glycolytic fibers [33], which is very similar to the plantaris muscle analyzed in the experiment of Armstrong et al. [32]. The two lowest exercise intensities in the investigation of Armstrong et al. [32] did not show glycogen depletion. It is essential to point out that both intensities correspond to the same intensity as our endurance protocol, which could justify this group’s lack of difference. Regarding the ET group, although the intensity used was the same as the END group, the animals ran until exhaustion, which depleted the muscle glycogen. The exercise intensity and volume may explain the lack of changes for the ST group. The ST (approximately 20 min) volume was lower compared to the other acute exercise sessions (ET ≈ 150 min, and CC ≈ 40 min). For the CC group, performing two types of exercise could justify the lower content of muscle glycogen.

Cambri et al. [34] submitted Wistar rats to one single swimming session at maximal lactate steady state and visualized a decrease in the soleus muscle’s glycogen after exercise. The maximal lactate steady state (MLSS) [35] is considered the highest exercise intensity at which blood lactate remains constant, and it is used to determine exercise intensity [35,36]. The physical exercise below and at the MLSS intensity correspond to the moderate-intensity domain [36]. For the END group, it is possible to speculate that the selection of an oxidative muscle, such as the soleus, for instance, would lead to similar results as those observed by Cambri et al. [34].

Reinforcing the ET’s high metabolic demand, this was the only exercise session able to increase the mRNA levels of *Prkaa1* and *Mtor* at 0 h, which are autophagy pathway classical modulators [7]. However, none of the physical exercise protocols were able to modify the autophagy genes at 0 h. In contrast, Vainshtein et al. [37] observed an increase in mRNA levels of *Map1lc3b* and *Sqstm1* in skeletal muscle immediately after an acute exhaustive exercise protocol. Our ET group ran at a lower intensity than Vainshtein et al. [37] (13.9 ± 1.7 m/min versus 30 m/min, respectively). Other authors have already highlighted that exercise intensity plays a fundamental role in skeletal muscle autophagy pathway modulation [38,39].

Most of the evaluated genes displayed significant differences at 6 h compared to 0 h for the same experimental groups and at 6 h among the experimental groups. These data reinforce that mRNA expression changes occurred from 3 to 12 h after cessation of the exercise stimulus [40]. Surprisingly, the main findings among the experimental groups at 6 h occurred for the ST group (i.e., increase in *Ppargc1a*, *Ulk1*, *Becn1*, *Map1lc3b*, and *Sqstm1*). Although the positive effects of strength exercise on the autophagy pathway have previously been described [21,41], this is the first investigation comparing different physical exercise protocols. The ST-induced autophagy activation may be related to the increase in *Ppargc1a* mRNA; Vainshtein et al. [37] showed that PGC-1α plays a role in acute exercise-induced autophagy.

Interestingly, the Beclin 1 was not modulated at 6 h. The ET and ST groups showed decreased Beclin 1 protein levels at 0 h compared to the CT group. The ATG5 protein levels were not modulated at 0 h and decreased at 6 h for the CT, END, and ET groups. Using murine gastrocnemius, Kim et al. [18] visualized a minorreduction in Beclin 1 protein levels at 0 h than the control group after an acute endurance exercise. In contrast to our findings, the Beclin 1 protein levels in Kim et al. [18] increased with time. These results reinforce that the exercise effects on the autophagic pathway are time-dependent [42].

The combination of strength and endurance acute physical exercise protocols modulated the LC3II and Sqstm1/p62 protein levels after colchicine treatment, indicating an increase in the markers of autophagy. Ranjbar et al. [22] verified a decrease in the LC3II/I ratio and an increase in Sqstm1/p62 in the tibialis of mice bearing the C26 colon carcinoma after a combined strength and endurance chronic physical exercise protocol. However, no treatments were used to measure the autophagic flux.

Autophagy participates in exercise-induced skeletal muscle performance improvement [43]. Thus, future investigations should compare these physical exercise protocols’ effects considering other skeletal muscle samples and time courses. It is essential to point out that the autophagic flux responses to chronic strength and concurrent exercise protocols have been investigated [44].

### 3.2. Effects of Acute Physical Exercise Protocols on the Heart

The lower glycogen concentrations of the ET and ST at 0 h display the cardiac metabolic challenges imposed by these acute exercise sessions. The mRNA levels of *Ppargc1a* and all autophagy genes were reduced at 0 h for the ET group. In agreement, Li et al. [45] demonstrated that a single exhaustive exercise session inhibited autophagy, leading to ischemia-hypoxia injury in rat myocardium. On the other hand, Liu et al. [46] verified that an exhaustive overload-exercise activated autophagy, linked to disrupted cardiomyocytes. In an elegant review, Sciarretta et al. [47] concluded that the functional significance of cardiac autophagy activation or inhibition in different conditions has yet to be clarified.

Acute and chronic excessive exercise models increased the cytokine levels (i.e., interleukin-6, IL-6, and tumor necrosis factor-alpha, TNFα) in the serum and hearts of mice [48,49,50]. Some studies demonstrated that in inflammatory conditions, the PGC1α levels are downregulated, reporting a pivotal role of proinflammatory cytokines in modifying the PGC1α levels [51,52,53]. For instance, after being treated with TNFα, cardiac cells demonstrated a decrease in PGC1α levels [51]. In the present study, for the ET group, the *Ppargc1α* mRNA levels were lower than the other groups, probably because this type of exercise upregulated the cytokine levels. Although the cytokine levels were not evaluated after exhaustive exercise in the present study, this response is already well described in the literature [48,49].

In addition, the mRNA levels of *Ppargc1a*, *Map1lc3b*, and *Sqstm1* were reduced at 0 h for the ST group. Compared to 0 h, only the mRNA levels of *Prkaa1* were modulated for all experimental groups at 6 h. The mRNA levels of *Ulk1* and *Atg5* were up-regulated in response to some experimental groups (*Ulk1* for END, ET, and ST, as well as *Atg5* for ET, ST, and CC) at 6 h compared to 0 h.

AMPK phosphorylates ULK1, which initiates the autophagy process. With the process occurring, the Atg5 is essential for phagophore formation [5]. One exercise bout generates an increase, although transient, in mRNA expression, and the temporal pattern is specific to a gene and the exercise challenge [40]. Based on this fact, we can infer that *Prkaa1* mRNA levels had a different pattern compared with *Ulk1* and *Atg5*, probably because it was up-regulated at another time point. Although all the genes analyzed in the present study are autophagy-related markers, each one is essential in a specific process in the autophagy pathway, which occurs at different times. Therefore, future studies should evaluate other time points of tissue collection to clarify the mRNA expression after exercise. To the best of our knowledge, this is the first investigation describing the acute effects of strength and concurrent exercise protocols on the cardiac autophagy pathway.

The protein levels of Beclin 1 were downregulated for the ET group at 0 h but without significant alteration at 6 h. Furthermore, the ET group showed lower values of ATG5 at 6 h. Yuan et al. [54] did not observe modifications in Beclin 1 in the heart tissue of rats after acute exhaustive treadmill running. Campos et al. [55] blocked the autophagic flux with chloroquine and visualized an increase in LC3II, Sqstm1/p62, and Beclin 1 in control animals, but not in heart failure animals. Herein, the ET group showed an increase in LC3II levels, but a decrease in Beclin 1, which could exacerbate cardiac function because alterations in autophagic activity can intensify or attenuate cardiac pathophysiology.

Autophagy flux assessment using lysosome inhibitors is fundamental to draw consistent conclusions [31,47]. Campos et al. [55] verified that the heart failure animal model presented a reduction in autophagic flux with an accumulation of autophagy-related markers and loss of sensitivity to chloroquine. After the chronic endurance exercise, the infarcted mice reestablished cardiac autophagic flux and increased the autophagic markers [55]. Investigations using rodents with knock-down genes related to the autophagosomes and lysosomes demonstrated the development of cardiac dysfunction, showing the relationship between autophagy and heart diseases [56,57]. Our findings revealed an increase in autophagy markers (i.e., Sqstm1/p62 protein levels after colchicine treatment) for the END and ST groups. Based on these data, future studies should verify the effects of endurance, strength, and a combination of both models in rodent models of heart failure as an alternative to recover the autophagic flux and improve cardiac failure.

### 3.3. Effects of Acute Physical Exercise Protocols on the Liver

The glycogen concentrations in the liver were not influenced immediately after the different acute physical exercise protocols. On the other hand, the mRNA levels of *Prkaa1*, *Ppargc1a*, *Ulk1*, and *Sqstm1* were up-regulated immediately after the ET session. Although this is the first investigation showing the acute effects of ET, ST, and CC protocols on the liver autophagy pathway, Kristensen et al. [58] have demonstrated that 60 min of running at 15 m/min and 10 degrees of inclination enhanced hepatic autophagy. According to these authors [58], elevated autophagy in the liver during or after exercise is needed for cellular component recycling and damaged protein removal. In agreement, Kwon et al. [59] showed that five days of moderate-intensity exercise activated hepatic autophagy.

The mRNA levels of *Prkaa1*, *Ppargc1a*, *Mtor*, *Becn1*, *Atg5*, and *Map1lc3b* displayed significant differences at 6 h compared to 0 h for the same experimental groups, reinforcing the alteration pattern of mRNA expression levels after acute exercise [40]. Regarding the mRNA expression levels at 6 h, the main differences were observed for the END (i.e., increase in *Ppargc1a* and *Ulk1*) and ET (i.e., increase in *Prkaa1*, *Ulk1*, and *Atg5*) protocols. For the ET group, the ATG5 protein levels were downregulated at 6 h, which is similar to what was visualized by Pinto et al. [60] after one bout of exhaustive exercise in the liver of control and IL-6 knockout mice.

Our autophagy flux experiment indicated no alterations of the LC3II and a reduction in the Sqstm1/p62 for the END and ET groups compared to the ST and CC groups. In addition, the CC group had higher Sqstm1/p62 than the ST group. Although other investigations regarding the effects of exercise on healthy liver demonstrated an increase in LC3II and a decrease in Sqstm1/p62, these authors did not use autophagy flux inhibitors (i.e., colchicine, chloroquine, etc.) [11,14,20,59,61,62]. Future investigations should evaluate these protocols’ chronic effects and their relationships with hepatic morphological adaptations, because Sqstm1/p62 deficiency was linked to diabetes and obesity development in adulthood [63]. In contrast, the deletion of Sqstm1/p62 suppressed liver dysfunction in autophagy-deficient mice [64].

**Figure 7:**
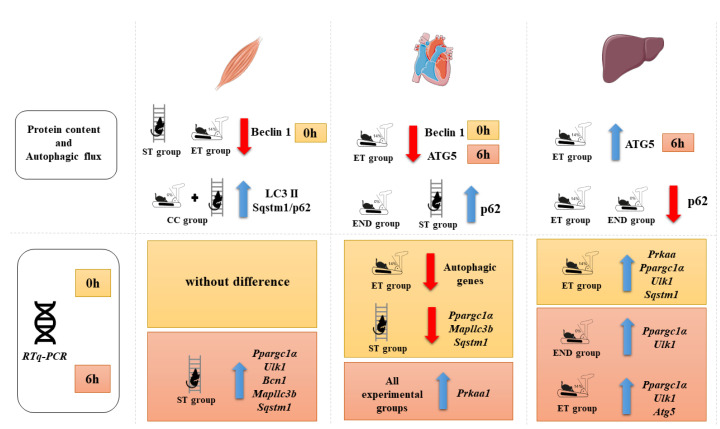
Schematic representation summarizing the main findings of the present study.

Our study’s most significant novelty was comparing different exercise models on autophagy markers in several metabolic tissues. In summary, the main changes in mRNA expressions in the gastrocnemius muscle occurred after 6 h for the ST group. In addition, the markers of autophagy were sensitive to the concurrent physical exercise protocol. The cardiac markers of autophagy were increased for the END and ST groups, highlighting these protocols’ potential to act as non-pharmacological agents to prevent and treat cardiovascular diseases. The hepatic protein levels of Sqstm1/p62 after colchicine treatment were reduced for the END and ET groups compared to the ST and CC groups. The physiological significance should be further addressed, because Sqstm1/p62 reduction may be linked to diabetes and obesity development or liver dysfunction improvement. It is possible to conclude that the ST and CC protocols induced better results for the evaluated metabolic tissues. Figure 7 summarizes the primary data of the present investigation.

One limitation of the current investigation was to measure the autophagy flux only by the Western blotting of LC3II and Sqstm1/p62 following colchicine treatment. Further studies should use more than one separate assay to evaluate the autophagic flux, such as flow and multispectral imaging cytometry, as well as fluorescence microscopy. The present investigation is critical to better comprehend the effects of different exercise modalities on autophagy signaling and regulation, however many questions still need to be investigated. Future studies should evaluate how exercise intensity and duration may impact the crosstalk between the tissues and which exercise model is more effective for improving specific pathologies in these tissues.

## 4. Materials and Methods

### 4.1. Animals

Six-week-old male C57BL/6 mice from the Central Animal Facility of the Ribeirão Preto campus of the University of São Paulo (USP) were kept in sterile micro-insulators (three animals per cage) in a ventilated rack (INSIGHT, Ribeirão Preto, SP, Brazil) with controlled temperature (22 ± 2 °C) on a 12:12 h light–dark normal cycle. Food (Purina chow) and water were provided ad libitum. The methods were carried out following the Brazilian College of Animal Experimentation (COBEA) and were approved by the University of Sao Paulo (I.D. 2017.5.30.90.8). Mice were divided into five experimental groups: Control (CT; sedentary), Endurance (END; submitted to the endurance protocol), Exhaustive (ET; submitted to the exhaustive protocol), Strength (ST; submitted to the strength protocol), and Concurrent (CC; submitted to the concurrent protocol). The sample size (*n*) for each experiment is available in the figure legends.

### 4.2. Experimental Procedures

After two weeks of ambient adaption, eight-week-old male C57BL/6 mice started the exercise adaptation. Mice from the END, ET, and CC groups were submitted to a 1 week adaptation period on a treadmill (INSIGHT, Ribeirão Preto, SP, Brazil) for five days, 10 min/day, at a speed of 6 m.min^−1^. The mice from the ST group were submitted to a 1 week adaptation period of ladder-climbing (INSIGHT, Ribeirão Preto, SP, Brazil) with (i.e., two days) and without (i.e., three days) external load [65]. The ladder was 1110 mm high, with an 80° incline and 85 steps, with a distance of 6 mm between each rung.

#### 4.2.1. Incremental Load Test

The incremental load test started at an initial velocity of 6 m/min, at a 0% incline for the END and CC groups, and a −14% incline for the ET group, with increments of 3 m/min every 3 min until voluntary exhaustion. The exhaustion velocity (EV) was used to prescribe the exercise intensities for the END, ET, and CC groups.

#### 4.2.2. Acute Physical Exercise Protocols

**Figure 8:**
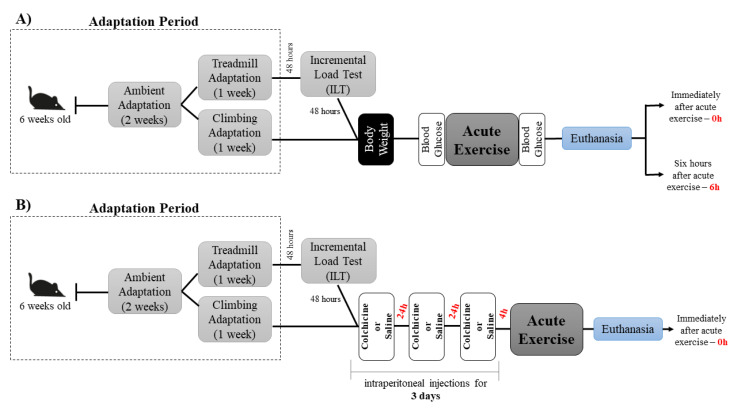
Schematic representation of the experimental procedures (**A**,**B**).

The END and ET exercise protocols consisted of treadmill running in which the intensity was obtained after the incremental load test. The END and ET groups ran at 60% of the EV. The END group ran for 60 min at a 0% incline [35], while mice from the ET group ran until exhaustion at a −14% incline [66]. The ST group performed the strength protocol, which consisted of ladder-climbing. To warm up, the mice performed one climb without any load. After that, an external load was attached to the tail, corresponding to 75% of their body weight. The mice performed ten climbs with a 1 min recovery between each climb [65]. Mice from the CC group performed the strength protocol first, followed by the endurance protocol. The animals started with one climb without an external load to warm up. After that, mice climbed five sets with 75% of body weight attached to their tails, with a 1 min recovery between each climb. After finishing the strength protocol, the animals ran at 60% of the EV at a 0% incline for 30 min. The animals were euthanized immediately (0 h) and six hours (6 h) after the acute physical exercise protocols. Figure 8A,B illustrate the schematic representation of the experimental procedures.

### 4.3. Glucose Levels

Blood from the tail tip was collected, and glucose levels were measured before and immediately after the acute physical exercise protocols using a glycemic monitoring system (ACCU-CHEK Active model, Roche, Santo André, SP, Brazil).

### 4.4. Tissue Extractions

Immediately (0 h) and six hours (6 h) after the acute physical exercise protocols, the animals were anesthetized by an intraperitoneal administration of xylazine (10 mg/kg body weight) and ketamine (100 mg/kg body weight). As soon as the loss of pedal reflexes confirmed the effect of anesthesia, the gastrocnemius, heart (left ventricle isolation), and liver samples were removed, washed with saline, and used for glycogen analysis, reverse transcription-quantitative polymerase chain reaction (RTq-PCR), and immunoblotting.

### 4.5. Glycogen Concentrations

The gastrocnemius, heart, and liver glycogen concentrations were measured as described by Dubois et al. [67].

### 4.6. Reverse Transcription-Quantitative Polymerase Chain Reaction (RTq-PCR)

The samples were collected and immediately placed in RNA later solution (AMBION, Foster City, CA, USA) and stored at −80 °C until RNA isolation. All procedures were performed under standard RNase-free conditions to avoid exogenous RNase contamination.

Total RNA was extracted with Trizol (INVITROGEN, Carlsbad, CA, USA). The cDNA was synthesized with 1000 ng of total RNA using the High-Capacity cDNA Reverse Transcription Kit (APPLIED BIOSYSTEMS, Foster City, CA, USA), according to the manufacturer’s instructions. Quantitative real-time PCR was performed on the ViiA7 Real-Time PCR System (APPLIED BIOSYSTEMS, Foster City, CA, USA) to analyze the relative mRNA expression of the following genes: *Prkaa1* (protein kinase, AMP-activated, alpha 1 catalytic subunit), *Ppargc1a* (peroxisome proliferative activated receptor, gamma, coactivator 1 alpha), *Mtor* (mechanistic target of rapamycin kinase), *Ulk1* (unc-51-like kinase 1), *Becn1* (Beclin 1), *Atg5* (autophagy-related 5), *Map1lc3b* (microtubule-associated protein 1 light chain 3 beta), and *Sqstm1* (sequestosome 1/p62). Table 2 shows the design of the primers.

The amplification reactions (10 µL final volume) were performed in duplicate with the following reagents: 5 µL 2× Power Sybr Master Mix (THERMO FISHER SCIENTIFIC, Wilmington, DE, USA), 1 uL primer forward, 1 µL primer reverse, 1 µL cDNA diluted in 1:10 and 2 µL of H_2_O. The reference biological group was the control group 0 h for the exercise groups at 0 h (i.e., CT, END, ET, ST, and CC—0 h), and the reference biological group was the control group 6 h for the exercise groups at 6 h (i.e., CT, END, ET, ST, and CC—6 h). *Gapdh* was used as a reference gene for the normalization of the data. Each amplification reaction occurred in the standard cycling in the following cycles: 10 min at 95 °C and a further 40 cycles with 15 s at 95 °C and 1 min at 60 °C. Relative quantification was calculated by the 2 −ΔΔCT method using the Thermo Fisher Cloud Software, RQ version 3.7 (LIFE TECHNOLOGIES CORPORATION, Carlsbad, CA, USA).

### 4.7. Markers of Autophagy

Mice were treated with intraperitoneal injections of colchicine (0.4 mg/kg/day; AB120663, ABCAM, Cambridge, UK) or vehicle (0.9% saline) for 3 consecutive days. The final injection was performed 4 h before the acute physical exercise protocols [44,68,69,70]. The colchicine treatment inhibits autophagosome degradation, inducing an increase in the levels of LC3II and/or Sqstm1/p62 [71]. The mean colchicine values were subtracted from the vehicle values of corresponding protocols (i.e., mean CT with colchicine-mean CT with saline) for autophagic flux calculation. Immediately after the acute physical exercise protocols, the animals were anesthetized by an intraperitoneal administration of xylazine (10 mg/kg body weight) and ketamine (100 mg/kg body weight). As soon as the loss of pedal reflexes confirmed the effect of anesthesia, the gastrocnemius, heart (left ventricle isolation), and liver samples were removed, washed with saline, and used for the immunoblotting technique.

### 4.8. Immunoblotting Technique

The immunoblotting technique was performed as previously described [50,72]. The antibodies used were Beclin 1 (#3738), ATG5 (#12994), LC3B (#2775), Sqstm1/p62 (#23214), and GAPDH (#2118) from CELL Signaling Technology (Danvers, MA, USA). All the primary antibodies were utilized at a dilution of 1:1000, and the secondary antibody (#7074s) from CELL Signaling Technology (Danvers, MA, USA) was used at a dilution between 1:10,000 and 1:20,000. GAPDH was used as a reference protein for the normalization of data. Ponceau S solution (#P7170) from SIGMA-ALDRICH (St. Louis, MO, USA) was used to verify total protein stain for immunoblotting normalization [73]. Images were acquired by the C-Digit Blot Scanner (LI-COR, Lincoln, Nebraska, USA) and quantified using the software Image Studio for C-DiGit Blot Scanner.

### 4.9. Statistical Analysis

Results are expressed as the mean ± standard error of the mean (SE). The Shapiro–Wilk *W*-test was used to verify data normality, and Levene’s test was used to test the homogeneity of variances. When normality or homogeneity was confirmed, one-way or two-way analysis of variance (ANOVA) was used to examine the differences between the experimental groups. All statistical analyses were two-sided, and the significance level was set at *p* ≤ 0.05. Statistical analyses were performed using the software SPSS v.20.0 for Windows (IBM, Chicago, IL, USA).

## Figures and Tables

**Table 1:** Body weight, basal glucose, post-exercise glucose, and delta variation. Data correspond to the mean ± standard error of the mean (SE) of *n* = 5 mice. CT: sedentary mice; END: mice submitted to the endurance protocol; ET: mice submitted to the exhaustive protocol; ST: mice submitted to the strength protocol; CC: mice submitted to the concurrent protocol. ^a^ *p* ≤ 0.05 vs. basal glucose for the same group; ^b^ *p* ≤ 0.05 vs. delta variation of the ET group.

Parameters	CT	END	ET	ST	CC
Body weight (g)	23.3 ± 0.5	22.9 ± 0.4	22.7 ± 0.8	23.6 ± 0.5	21.7 ± 0.43
Basal glucose (mg/dL)	157.4 ± 5.5	164.4 ± 5.6	193.8 ± 3.2	173.6 ± 7.9	154.6 ± 6.1
Post-exercise glucose (mg/dL)	-	147.8 ± 24.1	109.6 ± 12.2 ^a^	187.2 ± 12.3	126.0 ± 10.4 ^a^
Delta variation (%)	-	10.7 ± 12.79	−43.3 ± 6.6	−9.4 ± 10.1 ^b^	−18.6 ± 5.3

**Table 2:** The design of the primers.

Gene	Forward	Reverse
*Prkaa1*	CCAGGTCATCAGTACACCATCT	TTTCCTTTTCGTCCAACCTTCC
*Ppargc1a*	GAGTTGAAAAAGCTTGACTGGC	CAGCACACTCTATGTCACTCCA
*Mtor*	CCACGTGGTTAGCCAGACT	TAGCGGATATCAGGGTCAGGA
*Ulk1*	AACATCCGAGTCAAGATTGCTG	ATAATGACCTCAGGAGCCATGT
*Becn1*	AGGAACTCACAGCTCCATTACT	CTCTCCTGAGTTAGCCTCTTCC
*Atg5*	GCTTTTGCCAAGAGTCAGCTAT	AACCAATTGGATAATGCCATTTCAG
*Map1lc3b*	AGATAATCAGACGGCGCTTG	TCGTACACTTCGGAGATGGG
*Sqstm1*	ACAGCCAGAGGAACAGATGG	GTAGAGACTGGAGTTCACCTGTA
*Gapdh*	AAGAGGGATGCTGCCCTTAC	CGGGACGAGGAAACACTCTC

## Data Availability

Data is contained within the article or supplementary material.

## Supplementary Materials

Supplementary materials can be found at https://www.mdpi.com/1422-0067/22/5/2635/s1.

## Abbreviations

ATG	Autophagy-related gene
BCL-2	B cell lymphoma 2
LC3	Microtubule-associated protein
END	Endurance exercise
ET	Exhaustive exercise
ST	Strength exercise
CC	Concurrent exercise
MLSS	Maximal lactate steady state
EV	Exhaustion velocity
