# Supplementary material for: Impact of Different Physical Exercises on the Expression of Autophagy Markers in Mice
Source: Int J Mol Sci. 2021 Mar 5;22(5):2635. doi: 10.3390/ijms22052635 (PMC7962017; doi:10.3390/ijms22052635)
Supplement: Supplementary file 1 [file ijms-22-02635-s001.zip › Supplementary file 2.pdf]

Article

Acute impact of different physical exercise models in autophagy flux of mice metabolic organs

Ana P. Pinto[1], Alisson L. da Rocha[1], Bruno B. Marafon[2], Rafael L. Rovina[2], Vitor R. Muñoz[3], Lilian E. C. M. da Silva[4], José R. Pauli[3], Leandro P. de Moura[3], Dennys E. Cintra[3], Eduardo R. Ropelle[3], and Adelino S. R. da Silva[1,2].

GASTROCNEMIUS – Autophagic Flux

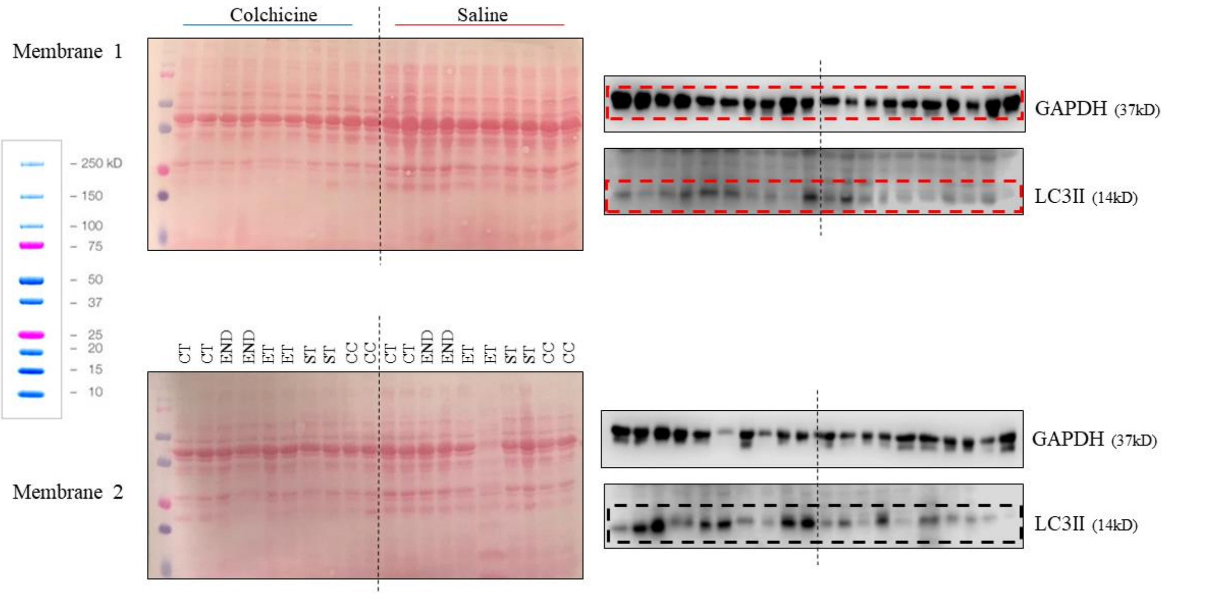

GASTROCNEMIUS – Autophagic Flux

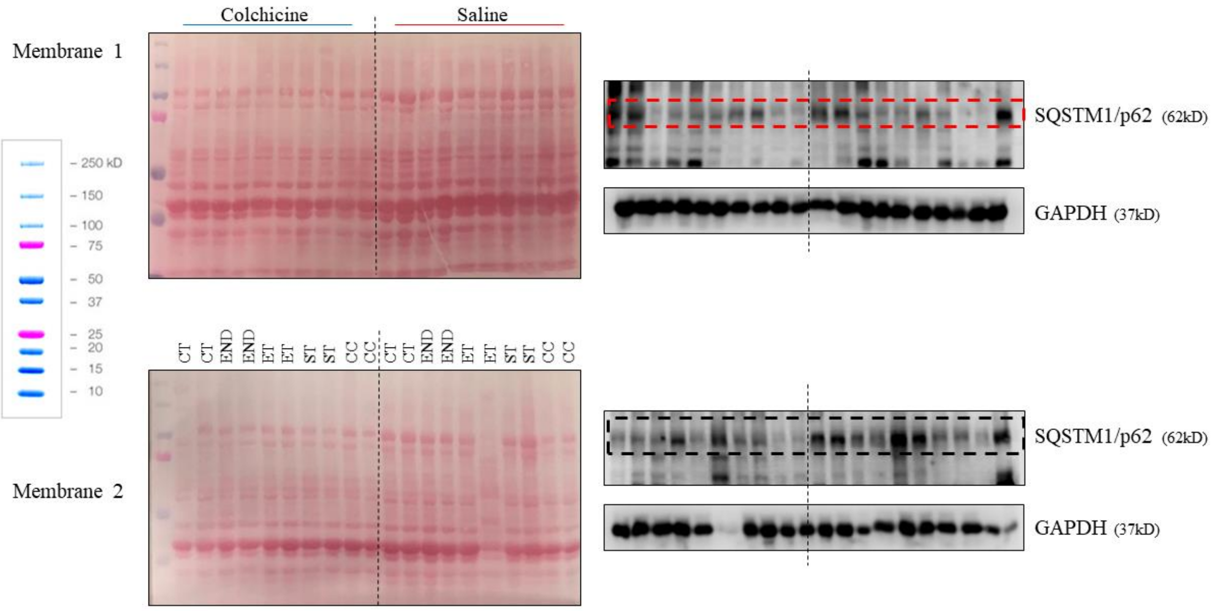

HEART – Autophagic Flux

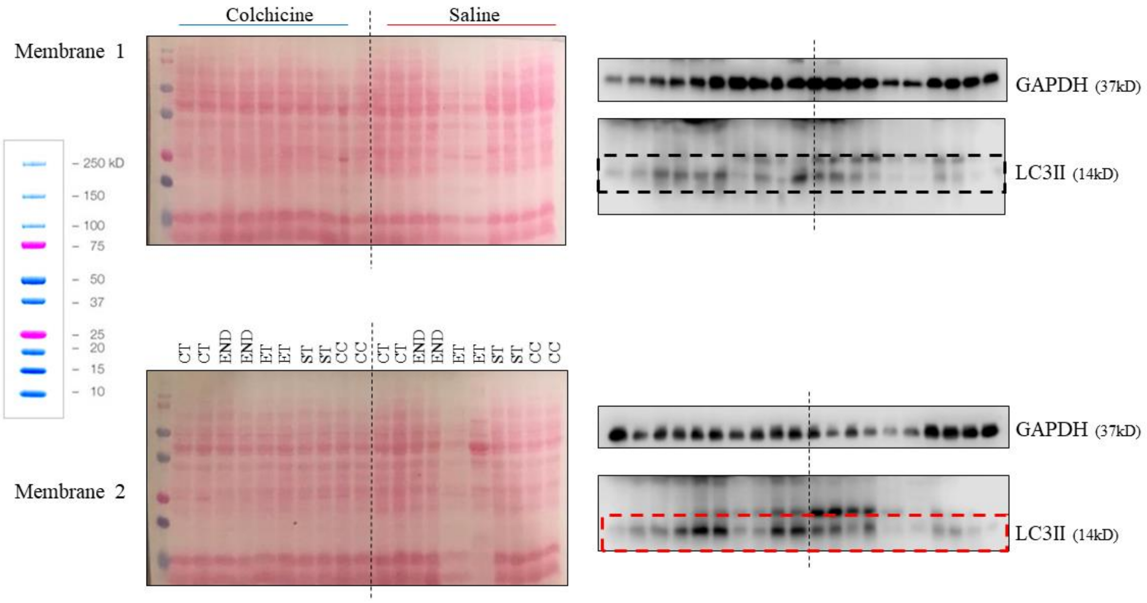

HEART – Autophagic Flux

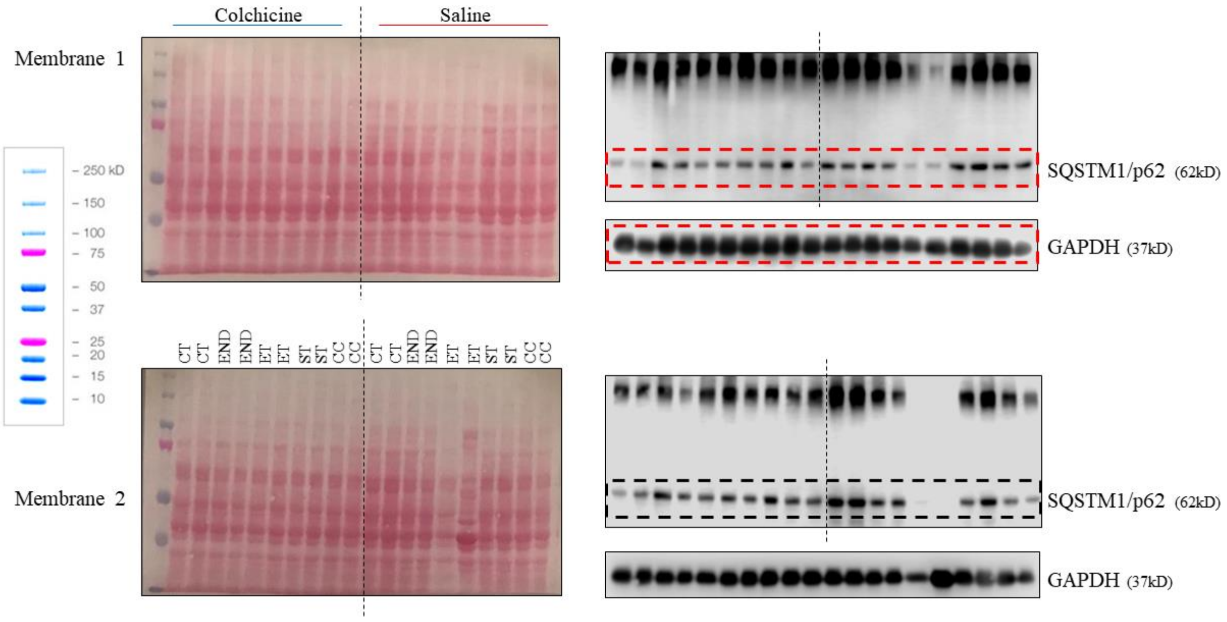

LIVER – Autophagic Flux

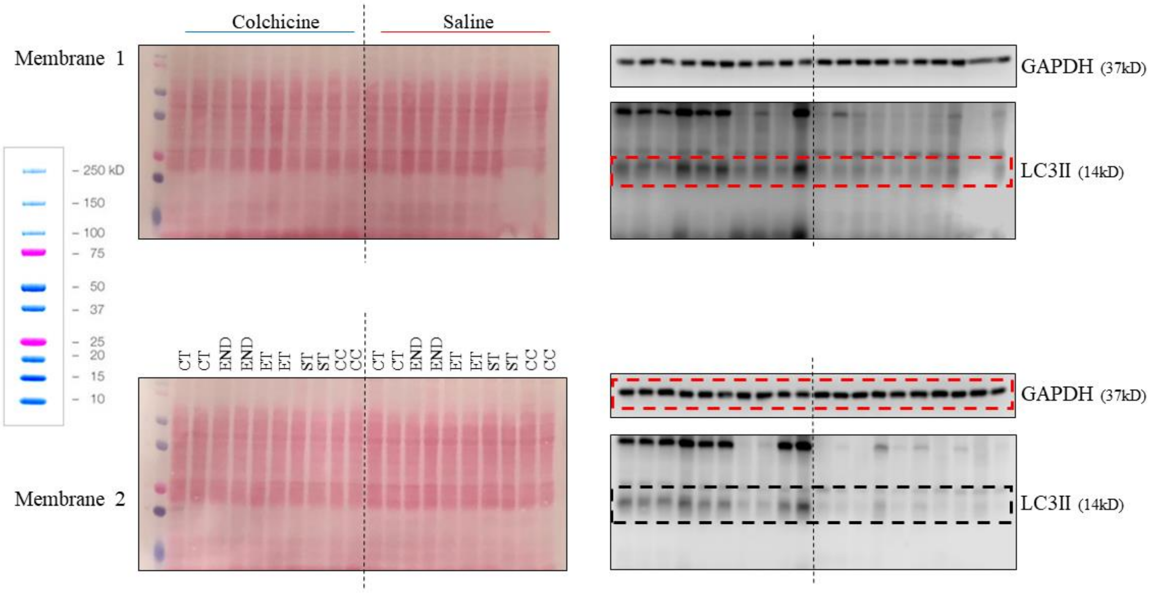

LIVER – Autophagic Flux

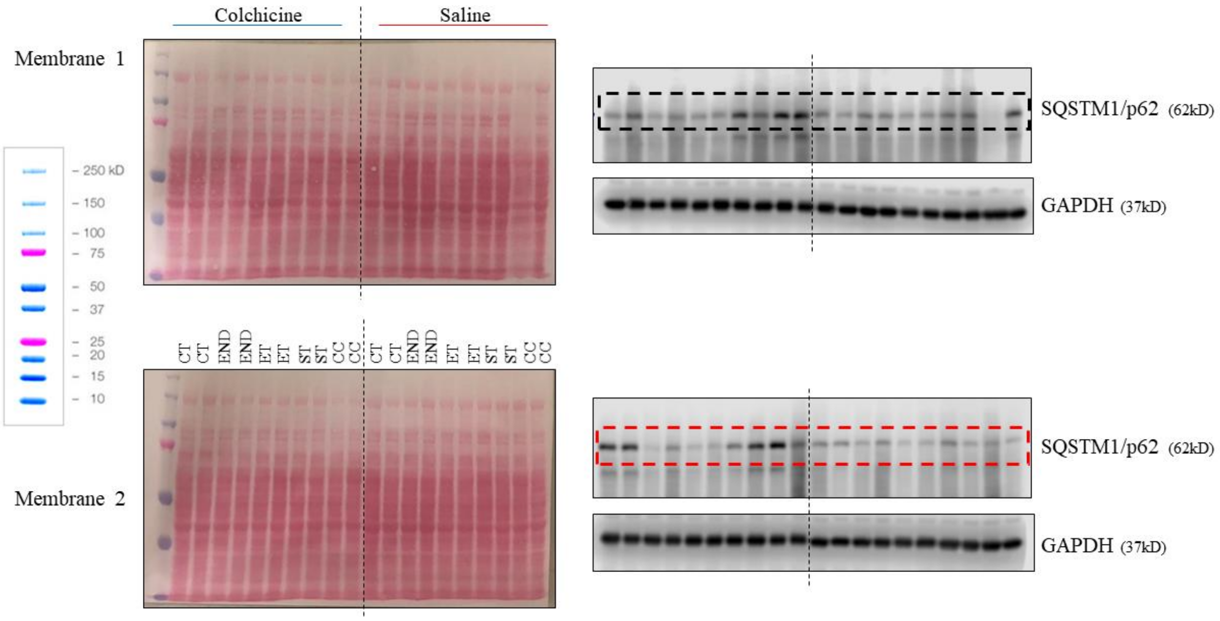

Supplementary file 2 - Ponceau with full bands and kD. Gastrocnemius, heart and liver protein contents of LC3II and SQSTM1/p62. Data correspond to the mean ± SE of n=5 mice/group. The dotted red squares correspond to the representative bands in figure 6.
